# Predicting Transmural Lesion Formation and Steam‐Pop Occurrence During Bipolar Ablation—Ex Vivo Porcine Model

**DOI:** 10.1002/joa3.70337

**Published:** 2026-04-07

**Authors:** Hisaki Makimoto, Masashi Kamioka, Tomonori Watanabe, Hiroaki Watanabe, Toshiya Kakurai, Naoki Suda, Takafumi Okuyama, Ayako Yokota, Takahiro Komori, Tomoyuki Kabutoya, Asuka Makimoto, Yasushi Imai, Kazuomi Kario

**Affiliations:** ^1^ Division of Cardiovascular Medicine, Department of Medicine Jichi Medical University School of Medicine Shimotsuke Tochigi Japan; ^2^ Clinical Research Center Jichi Medical University Shimotsuke Tochigi Japan

**Keywords:** bipolar ablation, catheter ablation, steam pop, transmurality, ventricular tachycardia

## Abstract

**Background:**

Bipolar radiofrequency (RF) ablation can create deeper myocardial lesions than unipolar ablation, yet its optimal settings remain undefined.

**Objective:**

To develop and validate predictive models for lesion transmurality and steam‐pop occurrence during bipolar ablation.

**Methods:**

Ex vivo bipolar ablation was applied to porcine myocardium (5–20 mm thickness) with catheters placed bilaterally at 45‐degree and 10‐g contact‐force. RF power (20–50 W) and duration (20–180 s) were systematically varied. Generalized linear models (GLM) were trained on 194 applications to predict transmurality and steam‐pop from RF energy, tissue thickness, initial bipolar impedance, 5‐s impedance drop (absolute and percentage [PercentImpDrop5]), and RF duration; 111 independent applications served as the validation.

**Results:**

Training yielded 95 transmural lesions (49%) and 11 steam‐pops (5.7%). For transmurality, the model incorporating RF energy, RF duration, initial impedance, and tissue thickness achieved an area under the receiver‐operating characteristics curve (AUC) of 0.95 (95% CI 0.91–0.99) with 88% sensitivity and 100% specificity. Omitting tissue thickness markedly degraded performance (AUC 0.68; DeLong, *p* = 0.003). For steam‐pop, the model combining RF energy and PercentImpDrop5 showed the best discrimination (AUC 0.90 [0.82–0.97], sensitivity 84%, specificity 90%); notably, PercentImpDrop5 alone achieved comparable accuracy (AUC 0.89).

**Conclusion:**

Tissue thickness is the dominant determinant of transmural lesion formation, whereas early impedance drop serves as a reliable real‐time indicator of steam‐pop risk during bipolar RF ablation. These algorithms may help standardize bipolar ablation protocols by enabling prospective titration of energy delivery based on tissue characteristics and intraprocedural impedance monitoring.

## Graphical abstract

Using ex vivo porcine myocardium, we developed predictive models for bipolar radiofrequency ablation: tissue thickness predicted transmurality (AUC 0.95) and early impedance drop indicated steam‐pop risk (AUC 0.89–0.90). These algorithms may standardize ablation protocols through tissue‐based energy titration and real‐time impedance monitoring.

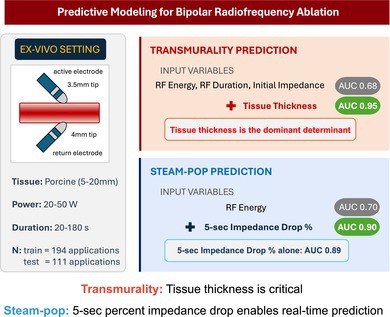

## 1 Introduction

Bipolar ablation has been reported to create deeper myocardial lesion as compared to unipolar ablation; however, there is currently no standardized strategy for bipolar ablation settings such as energy output and ablation time. During ablation procedures within the ventricles, the created lesion depth is a critical concern. To achieve deeper lesions, it is common to increase the radiofrequency (RF) energy output and the duration of ablation [1, 2]. However, these strategies are associated with an increased risk of steam pops [3, 4, 5]. Steam pops can cause unexpected physical damage to the myocardium, resulting in intraoperative complications [6].

Bipolar ablation has been developed as a therapeutic option to ablate arrhythmic foci deep within the myocardium [7]. This technique enables transmural lesions formation by employing RF energy between ablation catheters positioned on opposite sides of the myocardium. However, in clinical practice, it is not possible to visually confirm whether truly deep, transmural lesions have been formed. Moreover, in bipolar ablation, the occurrence of steam pops can also lead to potential complications.

Therefore, there is a need to identify ablation settings that can create transmural lesions while avoiding the occurrence of steam pops.

The aim of the present study is to test our hypothesis that both transmural lesion formation and steam‐pop occurrence during bipolar ablation can be predicted by the settings of bipolar ablation and the myocardial tissue characteristics such as tissue thickness and periprocedural impedance dynamics. We intended to assess the created lesion in detail by ex vivo experiments using porcine myocardial tissues.

## 2 Methods

### 2.1 Ex Vivo Experimental Setup

The study protocol was approved by the Institutional Review Board (IRB) of Jichi Medical University. In its review, the IRB waived the need for formal approval from the Institutional Animal Care and Use Committee (IACUC). This decision was based on the use of postmortem porcine tissues obtained from a commercial supplier for human consumption, thereby ensuring that no animals were euthanized specifically for this study and adhering to the ‘Replacement’ principle of the Three Rs.

**FIGURE 1:**
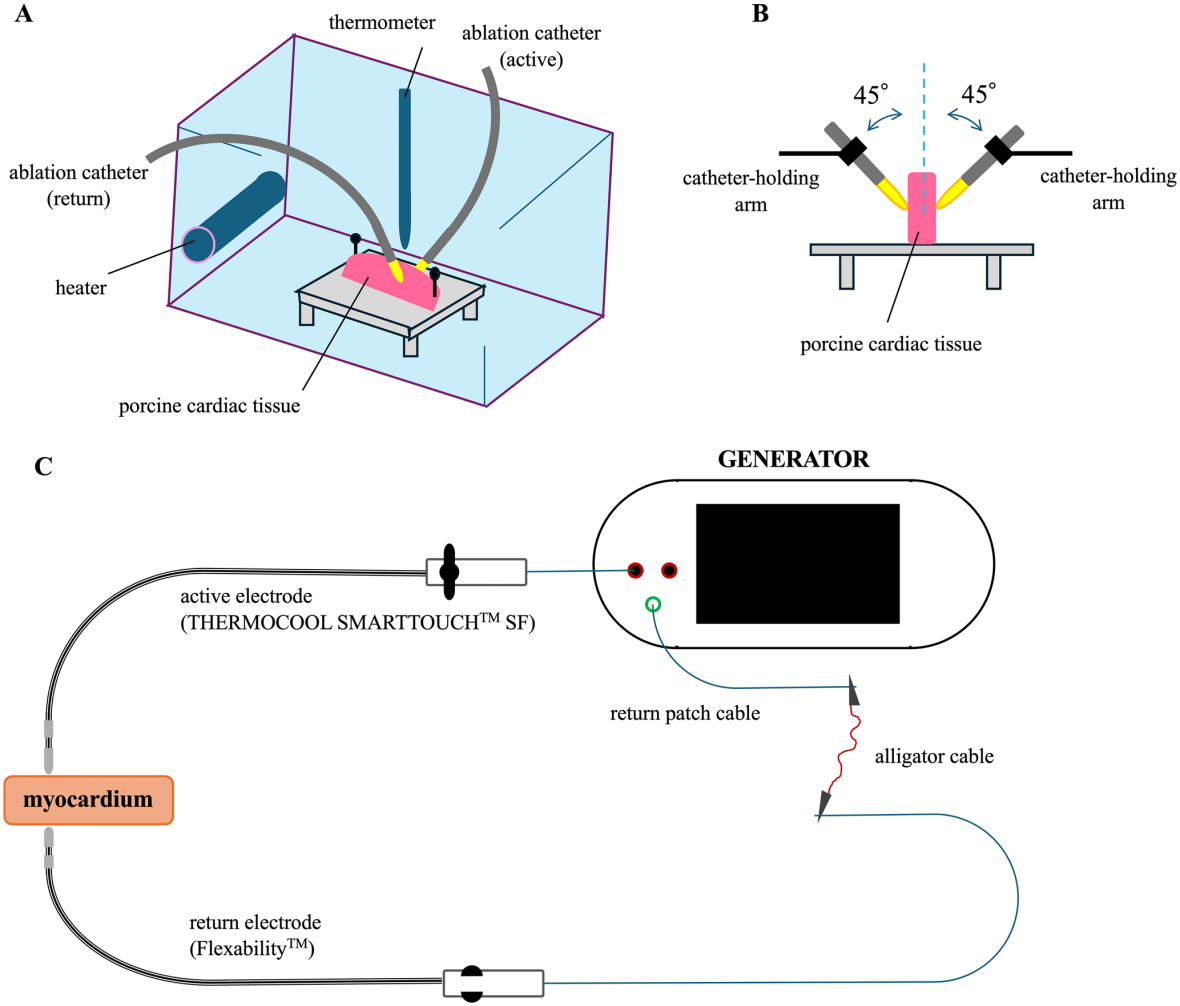
Experimental Setup. (A) The myocardial tissue samples were secured to an acrylic platform and submerged in a thermostatically controlled bath of physiological saline (0.9% NaCl) maintained at 36.5°C–37.5°C. (B) The active and dispersive (ground) ablation catheter electrodes were mounted on catheter‐holding arms and positioned at 45 degrees on opposite sides of the tissue. A target contact force of 10 g was continuously monitored on the SMARTTOUCH SF catheter (active electrode) using its contact force sensor, while the FlexAbility catheter (return electrode) was stabilized at a fixed position. An irrigation flow rate of 30 mL/min was applied throughout ablation. (C) The THERMOCOOL SMARTTOUCH SF catheter (Biosense Webster) was connected to the RF generator as the active (RF output) electrode. The FlexAbility ablation catheter (Abbott) was connected to the return (ground) pathway to establish a bipolar configuration. To achieve this configuration, the return electrode catheter was connected to the generator's dispersive (ground) port via a modified circuit. Specifically, the standard return patch cable was electrically bridged to the return electrode catheter cable using alligator clip connectors, thereby redirecting the return current from the dispersive skin patch to the intracardiac catheter tip. This setup represents an off‐label modification of the standard monopolar RF ablation circuit.

Using porcine cardiac tissues, we conducted ex vivo ablation experiments. The experimental setup is illustrated in Figure 1. For assessment of lesion formation, we performed RF ablation on freshly explanted swine hearts using the SMARTABLATE generator (Biosense Webster, Diamond Bar, CA, USA), in a bipolar catheter‐to‐catheter configuration with irrigated ablation catheters: a 3.5‐mm tip THERMOCOOL SMARTTOUCH SF Catheter (Biosense Webster, Diamond Bar, CA, USA) and a 4‐mm tip Flexability Ablation Catheter (Abbott, Abbott Park, IL, USA). The THERMOCOOL SMARTTOUCH SF Catheter was connected to the generator as the active (RF output) electrode, and the Flexability Ablation Catheter was connected as the return (ground) electrode, such that RF current was delivered primarily through the myocardial tissue between two catheter tips. Because the FlexAbility catheter was connected to the indifferent (return) port of the generator, its tip temperature was not recorded by the system. Although the THERMOCOOL SMARTTOUCH SF catheter incorporates a thermocouple, temperature readings from open‐irrigated tips substantially underestimate true tissue temperature due to saline cooling; therefore, catheter‐tip temperature was not used as a study parameter.

We selected the left ventricular septum with a thickness of at least 15 mm for dissection. The dissected myocardial mass was then subdivided into pieces measuring approximately 10, 15, and 20 mm. These tissue samples were affixed to a platform and submerged in a thermostatically controlled bath of physiological saline (0.9% NaCl) maintained at 36.5°C–37.5°C. The ablation catheters, both active and ground, were positioned at 45° on opposite sides of the tissue.

Both catheters were stabilized using a mechanical holder to maintain a constant catheter‐tissue geometry during RF delivery. A target contact force of 10 g was applied and continuously monitored on the THERMOCOOL SMARTTOUCH SF catheter (active electrode) side using its contact force sensor. Because the Flexability catheter does not have a contact force sensor, its contact on the opposite side was standardized by fixation at a constant position with the holder; however, the exact contact force on the return electrode side could not be directly measured. Throughout ablation, an irrigation rate of 30 mL/min was applied.

For myocardial slices of 10 mm, 15 mm, and 20 mm thicknesses, we performed RF ablation using power settings of 20, 30, 40, and 50 W for durations of 20, 60, and 180 s. We monitored baseline generator impedance, maximum generator impedance drop, and the occurrence of audible steam pops. If an audible steam pop occurred, the RF application was stopped.

Key lesion parameters were macroscopically analyzed using a digital caliper. This analysis focused on estimating intramural lesion formation, with specific attention to maximal lesion depth and lesion surface diameters. Transmurality was determined as a macroscopically visible continuous lesion spanning between both catheters, assessed by two independent investigators (H.M. and M.K.). The transmurality rate was calculated as the percentage of transmural lesions within all lesions created using the respective approach.

### 2.2 Prediction Model Setup and Validation

Statistical analysis was conducted using Stata 18 (Stata Corp, USA) and python 3.90 environment on Mac OS 15. We developed separate generalized linear models (GLM) with a binomial family (logit link) to predict two binary outcomes: (1) lesion transmurality and (2) the occurrence of steam‐pop during RF application. Each model incorporated six candidate predictor variables: delivered RF energy (W), initial bipolar tissue impedance (Ω), RF duration (seconds), impedance drop in the first 5 s of ablation (ImpDrop5; Ω), 5‐s percentage impedance drop (PercentImpDrop5 = ImpDrop5 × 100/initial impedance; %) and tissue thickness (mm). Model training was performed on a development dataset, and coefficient estimates were obtained via maximum likelihood estimation. Subsequently, model performance was evaluated on an independent validation dataset using the area under the Receiver Operating Characteristic curve (AUC) as the primary measure of discrimination. For each outcome, we calculated the AUC on the validation set along with its 95% confidence interval (CI) to quantify model discrimination. The 95% CI for the AUC was derived using a nonparametric method (DeLong's algorithm) to estimate the standard error of the AUC.

The additional experiments for validation were conducted under the same condition with the experiments for model development. For efficient experiment planning, we performed the RF applications in which the conditions lied along the border line of steam‐pop occurrence based on the development dataset.

To assess the contribution of each variable to model performance, we employed an analysis analogous to Shapley value attribution from cooperative game theory [8]. Each predictor was treated as a “player” whose worth equals the average gain in validation AUC obtained by adding that variable to every possible subset of the other four predictors. For every predictor, we computed the AUC difference between the model that contained the variable and the otherwise identical model that did not, across all admissible subsets, and then averaged these differences. The resulting mean marginal AUC represents the variable's contribution to overall model performance and preserves Shapley's axioms of efficiency, symmetry, and additivity, yielding an interpretable, order‐independent measure of importance [9].

## 3 Results

### 3.1 Experiments for Model Development

#### 3.1.1 Transmurality of Created Lesions

**FIGURE 2:**
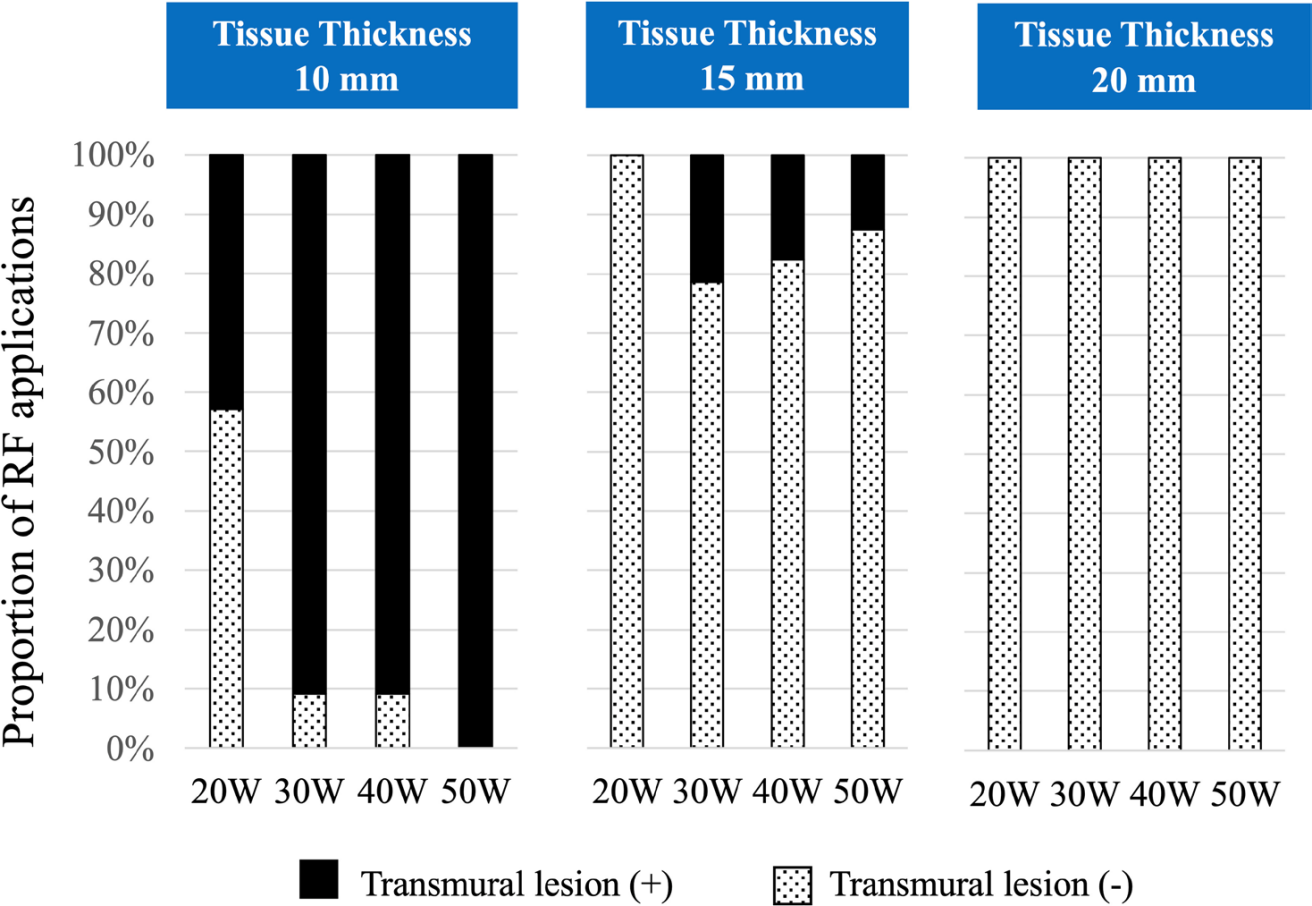
Transmurality of Created Lesions. Transmural lesions were noted in 95 out of 194 applications (49%). No transmural lesion formation was noted on the 20‐mm thickness tissues in 60 s even by 50 W RF energy. Transmural lesions were observed with RF energy of 30 W or more on most of the 10‐mm thickness tissues.

In total 194 bipolar RF applications were performed. Transmural lesions were noted in 95 out of 194 applications (49%, Figure 2).

No transmural lesion formation was noted on the 20‐mm thickness tissues in 60 s even by 50 W RF energy (Figure 2). Transmural lesions were observed with RF energy of 30 W or more on most of the 10‐mm thickness tissues. Prolonged duration of 180 s with 30 W or more accomplished the transmural lesions on the 15‐mm thickness tissues (Figure S1).

#### 3.1.2 Steam‐Pop

**FIGURE 3:**
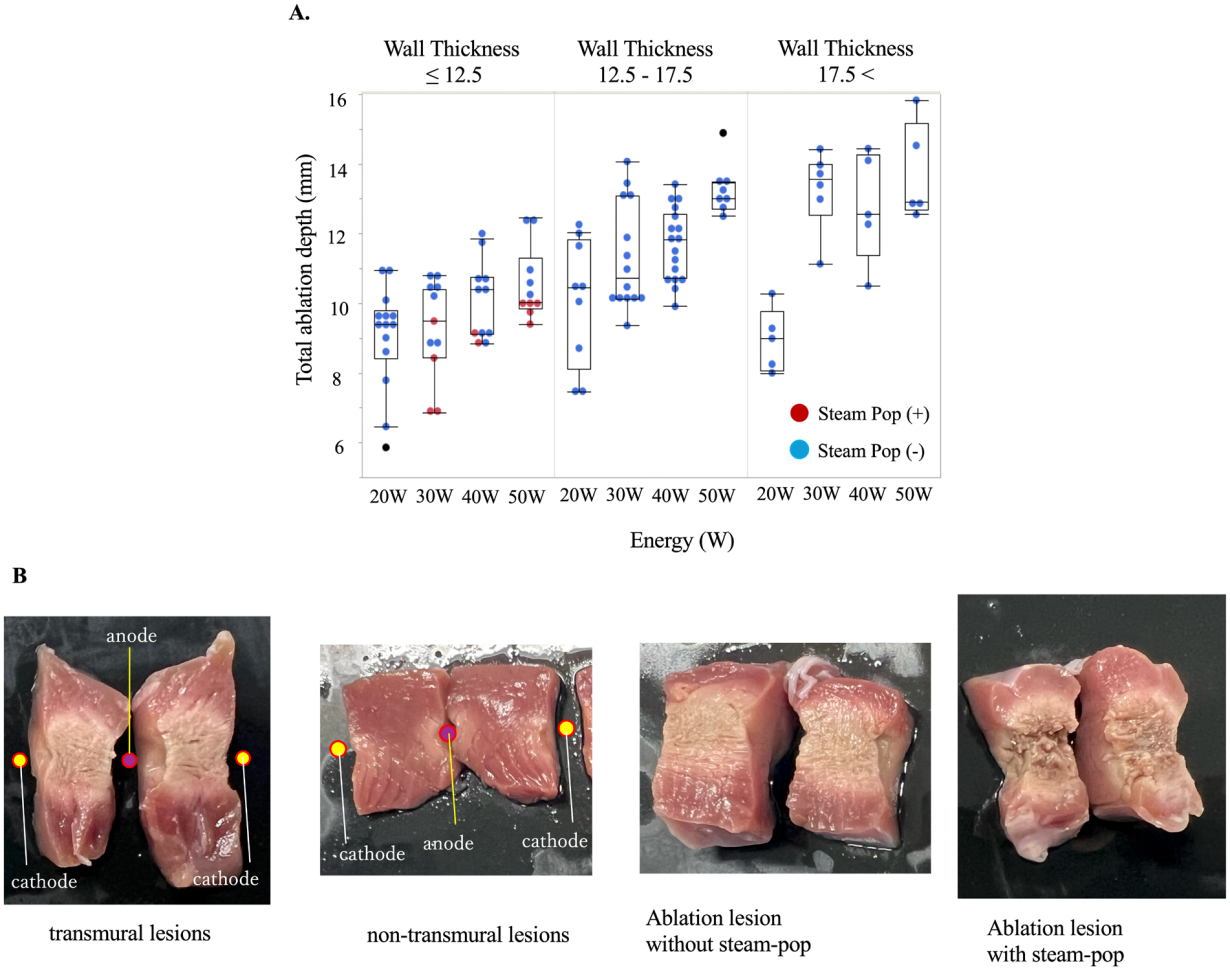
Ablation Lesion Formation and Steam Pop. (A) Audible steam‐pops were observed in 11 out of 194 applications (5.7%). (B) At the ablation sites where steam‐pop occurred, the myocardial tissue was torn, and tissue damage was evident.

Audible steam‐pops were observed in 11 out of 194 applications (5.7%, Figure 3A). At the ablation sites where steam‐pop occurred, the myocardial tissue was torn, and tissue damage was evident (Figure 3B).

**FIGURE 4:**
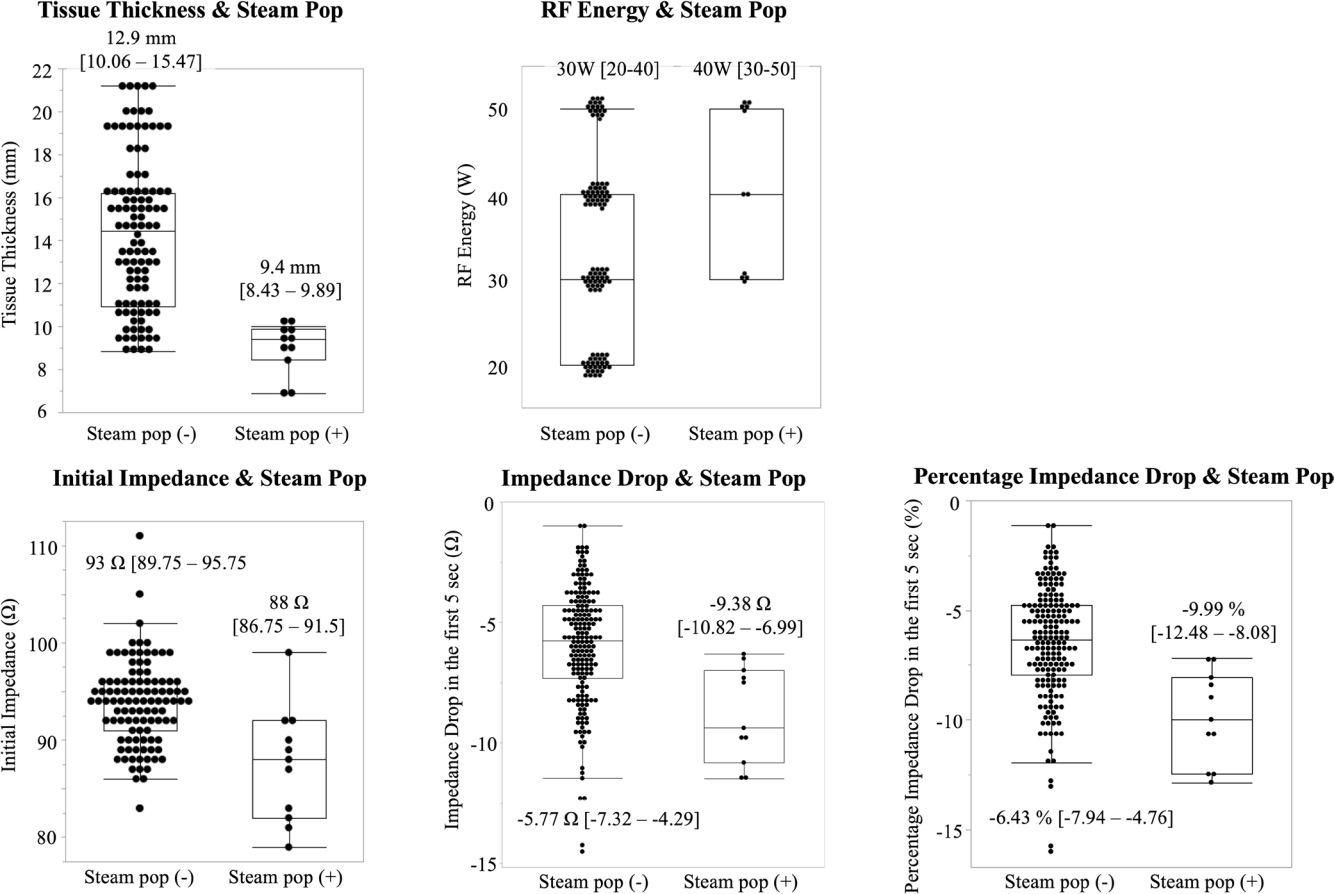
Steam‐pop Incidence and Tissue Characteristics. In applications with steam‐pop, RF energy was higher (median 40 W [q1‐q3: 30–50] vs. 30 W [20–40], *p* = 0.017), the initial impedance was lower (88 Ohm [86.75–91.5] vs. 93 Ohm [89.75–95.75], *p* = 0.0042), the tissue thickness was thinner (9.4 mm [8.43–9.89] vs. 12.9 mm [10.06–15.47], *p* < 0.0001), the ImpDrop5 was larger (−9.38 Ohm [−10.82 – −6.99] vs. −5.77 Ohm [−7.32 – −4.29], *p* = 0.0002), and the PercentImpDrop5 was larger (−9.99% [−12.48 – −8.08] vs. −6.34% [−7.94 – −4.76], *p* < 0.0001).

With regard to the applications with steam‐pop, the RF energy was higher (median 40 W [q1‐q3: 30–50] vs. 30 W [20–40], *p* = 0.017), the initial impedance was lower (88 Ohm [86.75–91.5] vs. 93 Ohm [89.75–95.75], *p* = 0.0042), the tissue thickness was thinner (9.4 mm [8.43–9.89] vs. 12.9 mm [10.06–15.47], *p* < 0.0001), and both the absolute and percentage impedance drops at 5 s were larger in applications with steam‐pop than those without (ImpDrop5: −9.38 Ohm [−10.82 to −6.99] vs. −5.77 Ohm [−7.32 to −4.29], *p* = 0.0002; PercentImpDrop5: −9.99% [−12.48 to −8.08] vs. −6.43% [−7.94 to −4.76], *p* < 0.0001, respectively; Figure 4).

### 3.2 GLM Model Development

Based on the above results, we utilized the following parameters for constructing the GLM construction: RF energy (W), RF duration (seconds), target tissue thickness (mm), initial tissue impedance (Ω), ImpDrop5 (Ω), and PercentImpDrop5 (%).

We built in total 63 models combining the above parameters for the transmurality and the steam‐pop occurrence, respectively (Tables 1, 2). These models were tested by the validation experiments below.

**TABLE 1:** Modeling of Generalized Linear Models for Transmurality.

RF energy	Initial impedance	RF duration	Impedance drop in 5 s	Impedance drop % in 5 s	Tissue thickness	auc	95% CI (Test)	auc_train	95% CI (Train)
✅	✅	✅			✅	0.95	0.908–0.992	0.942	0.913–0.971
✅		✅	✅		✅	0.95	0.910–0.990	0.957	0.932–0.981
✅		✅			✅	0.949	0.906–0.992	0.942	0.913–0.971
✅		✅		✅	✅	0.947	0.905–0.988	0.958	0.934–0.983
✅					✅	0.926	0.867–0.984	0.866	0.816–0.916
✅	✅	✅	✅		✅	0.922	0.862–0.981	0.961	0.938–0.984
✅	✅	✅		✅	✅	0.92	0.859–0.982	0.961	0.938–0.985
✅	✅	✅	✅		✅	0.92	0.862–0.979	0.961	0.937–0.984
✅		✅	✅	✅	✅	0.917	0.857–0.978	0.959	0.935–0.984
✅	✅				✅	0.91	0.844–0.975	0.863	0.812–0.913
					✅	0.887	0.821–0.953	0.808	0.749–0.867
		✅			✅	0.887	0.821–0.953	0.883	0.835–0.931
	✅	✅			✅	0.88	0.812–0.949	0.884	0.837–0.931
		✅	✅		✅	0.878	0.809–0.947	0.944	0.915–0.973
		✅		✅	✅	0.878	0.806–0.950	0.949	0.922–0.977
		✅	✅	✅	✅	0.878	0.801–0.955	0.957	0.932–0.982
	✅	✅	✅	✅	✅	0.873	0.791–0.955	0.958	0.934–0.983
	✅	✅	✅		✅	0.87	0.786–0.954	0.958	0.933–0.982
	✅	✅		✅	✅	0.87	0.790–0.951	0.958	0.934–0.982
	✅				✅	0.862	0.764–0.959	0.809	0.749–0.868
✅			✅		✅	0.839	0.755–0.922	0.892	0.847–0.937
✅				✅	✅	0.806	0.716–0.897	0.901	0.858–0.944
			✅		✅	0.793	0.697–0.889	0.889	0.843–0.934
				✅	✅	0.78	0.678–0.881	0.899	0.856–0.943
			✅	✅	✅	0.771	0.662–0.881	0.92	0.882–0.959
✅			✅	✅	✅	0.769	0.659–0.879	0.921	0.882–0.959
	✅			✅	✅	0.767	0.658–0.876	0.92	0.881–0.959
	✅		✅		✅	0.765	0.657–0.872	0.92	0.881–0.959
	✅		✅	✅	✅	0.765	0.656–0.874	0.92	0.881–0.959
✅	✅		✅	✅	✅	0.761	0.649–0.873	0.921	0.882–0.959
✅	✅		✅		✅	0.76	0.648–0.873	0.921	0.882–0.959
✅	✅			✅	✅	0.758	0.644–0.872	0.92	0.881–0.959
✅	✅	✅				0.68	0.483–0.877	0.719	0.647–0.791
✅	✅					0.678	0.480–0.875	0.71	0.637–0.782
	✅	✅	✅			0.678	0.570–0.785	0.894	0.849–0.939
	✅	✅	✅	✅		0.678	0.569–0.786	0.894	0.849–0.939
	✅		✅			0.675	0.566–0.785	0.885	0.837–0.933
	✅			✅		0.672	0.561–0.784	0.886	0.838–0.934
	✅	✅		✅		0.672	0.562–0.783	0.894	0.849–0.939
	✅		✅	✅		0.672	0.561–0.784	0.886	0.839–0.934
✅	✅			✅		0.67	0.546–0.794	0.898	0.853–0.942
			✅	✅		0.668	0.547–0.789	0.883	0.835–0.932
	✅					0.666	0.461–0.870	0.658	0.582–0.735
	✅	✅				0.666	0.461–0.870	0.674	0.598–0.750
✅	✅		✅			0.666	0.540–0.791	0.898	0.854–0.942
✅	✅		✅	✅		0.666	0.541–0.791	0.898	0.854–0.942
		✅	✅	✅		0.666	0.546–0.786	0.89	0.844–0.935
✅	✅	✅		✅		0.664	0.539–0.790	0.906	0.864–0.947
✅	✅	✅	✅			0.661	0.534–0.789	0.907	0.866–0.948
✅	✅	✅	✅	✅		0.661	0.534–0.789	0.907	0.866–0.948
✅			✅	✅		0.659	0.527–0.791	0.896	0.852–0.941
✅		✅	✅	✅		0.659	0.528–0.790	0.902	0.860–0.945
				✅		0.614	0.505–0.723	0.82	0.761–0.878
		✅		✅		0.614	0.505–0.723	0.832	0.777–0.887
✅				✅		0.602	0.480–0.725	0.828	0.771–0.885
✅		✅		✅		0.6	0.477–0.723	0.839	0.785–0.894
			✅			0.594	0.473–0.714	0.792	0.729–0.854
		✅	✅			0.594	0.473–0.714	0.806	0.747–0.866
✅			✅			0.586	0.455–0.717	0.792	0.730–0.855
✅		✅	✅			0.581	0.449–0.712	0.806	0.747–0.866
✅						0.562	0.443–0.681	0.632	0.556–0.708
✅		✅				0.562	0.443–0.681	0.64	0.563–0.717
		✅				0.5	0.500–0.500	0.501	0.426–0.576

**TABLE 2:** Modeling of Generalized Linear Models for Steam‐pop.

RF energy	Initial impedance	RF duration	Impedance drop in 5 s	Impedance drop % in 5 s	Tissue thickness	AUROC	95% CI (Test)	AUROC (train)	95% CI (Train)
✅				✅		0.895	0.823–0.968	0.859	0.780–0.939
✅		✅		✅		0.895	0.822–0.967	0.858	0.777–0.938
				✅		0.893	0.822–0.965	0.856	0.771–0.941
		✅		✅		0.893	0.822–0.965	0.857	0.772–0.942
✅	✅	✅	✅			0.882	0.805–0.959	0.916	0.840–0.991
✅	✅		✅			0.881	0.804–0.959	0.916	0.839–0.992
✅	✅	✅		✅		0.88	0.802–0.958	0.916	0.837–0.995
✅	✅			✅		0.878	0.799–0.956	0.915	0.836–0.993
	✅	✅	✅			0.877	0.798–0.956	0.91	0.832–0.988
	✅		✅			0.875	0.795–0.955	0.908	0.826–0.989
	✅	✅		✅		0.873	0.792–0.955	0.911	0.832–0.990
	✅			✅		0.872	0.790–0.953	0.907	0.820–0.993
			✅			0.869	0.791–0.946	0.832	0.737–0.926
		✅	✅			0.869	0.791–0.946	0.829	0.732–0.925
✅			✅			0.867	0.788–0.947	0.822	0.727–0.917
✅		✅	✅			0.867	0.788–0.947	0.826	0.733–0.919
✅	✅		✅	✅		0.867	0.785–0.949	0.915	0.833–0.996
✅	✅	✅	✅	✅		0.867	0.784–0.949	0.917	0.836–0.998
	✅		✅	✅		0.859	0.773–0.945	0.906	0.816–0.996
	✅	✅	✅	✅		0.859	0.773–0.945	0.908	0.821–0.995
✅		✅	✅	✅		0.844	0.748–0.939	0.915	0.828–1.000
✅			✅	✅		0.841	0.744–0.938	0.915	0.829–1.000
		✅	✅	✅		0.836	0.735–0.936	0.906	0.811–1.000
			✅	✅		0.833	0.731–0.934	0.905	0.805–1.000
✅	✅		✅	✅	✅	0.823	0.730–0.915	0.962	0.935–0.989
	✅		✅		✅	0.822	0.724–0.921	0.939	0.894–0.984
✅	✅		✅		✅	0.821	0.728–0.914	0.963	0.936–0.990
✅	✅			✅	✅	0.82	0.726–0.914	0.963	0.936–0.990
	✅			✅	✅	0.818	0.718–0.917	0.94	0.895–0.985
✅					✅	0.814	0.725–0.904	0.956	0.925–0.988
✅			✅	✅	✅	0.812	0.716–0.908	0.962	0.934–0.989
✅				✅	✅	0.809	0.718–0.899	0.956	0.925–0.988
✅	✅				✅	0.808	0.714–0.903	0.962	0.935–0.990
✅			✅		✅	0.806	0.715–0.897	0.957	0.926–0.988
✅		✅	✅		✅	0.805	0.712–0.897	0.961	0.933–0.988
✅		✅	✅	✅	✅	0.805	0.712–0.897	0.961	0.933–0.988
				✅	✅	0.803	0.704–0.902	0.932	0.892–0.972
✅		✅		✅	✅	0.803	0.709–0.896	0.96	0.933–0.988
	✅		✅	✅	✅	0.803	0.697–0.909	0.941	0.893–0.990
✅	✅	✅	✅		✅	0.801	0.708–0.894	0.961	0.933–0.988
✅	✅	✅		✅	✅	0.801	0.709–0.894	0.961	0.933–0.988
✅	✅	✅	✅		✅	0.801	0.708–0.894	0.961	0.933–0.988
✅		✅			✅	0.798	0.704–0.892	0.963	0.936–0.989
✅	✅	✅			✅	0.798	0.705–0.891	0.958	0.929–0.986
	✅	✅	✅		✅	0.798	0.697–0.899	0.95	0.918–0.982
			✅		✅	0.794	0.694–0.894	0.927	0.886–0.968
		✅		✅	✅	0.793	0.691–0.895	0.952	0.921–0.983
	✅	✅		✅	✅	0.793	0.691–0.896	0.95	0.918–0.982
			✅	✅	✅	0.792	0.681–0.902	0.942	0.893–0.991
		✅	✅		✅	0.788	0.685–0.891	0.952	0.921–0.983
	✅	✅	✅	✅	✅	0.787	0.682–0.891	0.951	0.919–0.983
		✅	✅	✅	✅	0.783	0.678–0.889	0.952	0.920–0.984
✅	✅					0.709	0.601–0.818	0.843	0.748–0.938
✅	✅	✅				0.709	0.601–0.818	0.854	0.760–0.948
✅						0.699	0.593–0.805	0.706	0.568–0.845
✅		✅				0.699	0.593–0.805	0.703	0.566–0.840
					✅	0.689	0.576–0.802	0.893	0.835–0.950
		✅			✅	0.689	0.576–0.802	0.925	0.876–0.974
	✅	✅			✅	0.688	0.575–0.802	0.927	0.878–0.976
	✅				✅	0.685	0.569–0.801	0.894	0.827–0.960
	✅					0.583	0.455–0.712	0.757	0.597–0.917
	✅	✅				0.583	0.455–0.712	0.768	0.612–0.924
		✅				0.500	0.500–0.500	0.443	0.396–0.490

### 3.3 Experiments for Model Validation

Target tissue thickness and delivered RF energy were the two experimental parameters which we could actively manipulate. Accordingly, we fitted a GLM to the development set using these predictors and plotted the resulting steam‐pop risk boundary in the plane (Figure S2). This boundary corresponds to the isopleth *Z* = 1 in the GLM‐derived equation, which marks a high steam‐pop probability of 92.6%.

$$\begin{array}{c} Z=0.0743\;\left(1+e^{\text{logit}}\right)\\ \;\left(\text{logit}=-4.9498+1.0729*\text{TissueThickness}-0.09030*\text{RFenergy}\;\right) \end{array}$$

The validation experiments were conducted under conditions chosen immediately adjacent to this risk boundary, with RF duration fixed at 60 s.

We performed 111 RF applications. Transmural lesions were noted in 102 out of 111 applications (92%) in the validation experiment. Steam‐pops were observed in 25 out of 111 applications (23%).

Using this validation dataset, we tested all the models built, except one model only using the RF duration because we fixed it at 60 s. The detailed test results are shown in Tables 1 and 2. The forest plots are also shown on the Figures S3 and S4.

The GLM, which utilized RF energy, tissue thickness, initial tissue impedance, and RF duration, performed the best in predicting transmural lesion formation (AUC 0.95 [0.91–0.99], sensitivity 88%, specificity 100%). However, the exclusion of tissue thickness from this model led to a significant decrease in its predictive performance (AUC 0.68 [0.48–0.88], sensitivity 64%, specificity 78%, *p* = 0.0030).

For steam‐pop prediction in validation data, the GLM utilizing RF energy and PercentImpDrop5 showed the best performance (AUC 0.90 [0.82–0.97], sensitivity 84%, specificity 90%). Removal of PercentImpDrop5 reduced the AUC by 0.20 (AUC 0.70 [0.59–0.81], sensitivity 76%, specificity 56%). Notably, the GLM utilizing PercentImpDrop5 alone achieved an AUC of 0.89 [0.82–0.97], which was comparable to the more complex model incorporating RF energy, initial bipolar impedance, RF duration, and ImpDrop5 (AUC 0.88 [0.81–0.96], sensitivity 80%, specificity 84%). When the absolute impedance drop (ImpDrop5) was removed from the latter model, the AUC decreased by 0.17 (AUC 0.71 [0.60–0.82], sensitivity 88%, specificity 47%).

The contribution of each variable to model performance was assessed using an analysis analogous to Shapley value attribution. For transmurality prediction, tissue thickness showed the largest contribution (0.22), followed by RF duration (0.047), initial impedance (0.023), and RF energy (0.017). Impedance drop parameters contributed minimally (ImpDrop5: −0.007; PercentImpDrop5: −0.006). For steam‐pop prediction, PercentImpDrop5 demonstrated the largest contribution (0.065), followed by ImpDrop5 (0.060), RF energy (0.036), and initial impedance (0.0087). RF duration (−0.0052) and tissue thickness (−0.024) showed negligible or negative contributions.

## 4 Discussion

The main findings of this study are as follows: (1) The transmurality of bipolar RF ablation can be predicted by utilizing RF energy, RF duration, target myocardial wall thickness, and preablation tissue impedance, (2) Prediction of steam‐pop occurrence during bipolar RF ablation can be achieved primarily by assessing the percentage impedance drop within 5 s (PercentImpDrop5), either alone or in combination with RF energy; alternatively, a model incorporating RF energy, preablation tissue impedance, and impedance drop within 5 s (ImpDrop5) achieves comparable accuracy. Notably, target myocardial wall thickness contributes significantly to predicting lesion transmurality, while the contribution of impedance‐related metrics is prominent in predicting steam‐pop occurrence.

To our knowledge, this is the first work to provide validated predictive algorithms for both efficacy and safety endpoints specific to bipolar ablation.

Korouth et al. [10] reported that increasing target myocardial wall thickness compromises transmurality during ex vivo bipolar experiments, highlighting the need to individualize energy delivery for thick myocardium. Consistent with the report, our model identified wall thickness as the single most influential predictor of lesion transmurality; removing this parameter reduced the AUC from 0.94 to 0.69.

An additional technical consideration is the asymmetry in electrode geometry in our bipolar setup: the active catheter had a 3.5‐mm irrigated tip (THERMOCOOL SMARTTOUCH SF) whereas the return catheter had a 4‐mm irrigated tip (FlexAbility). Differences in effective electrode surface area and tissue contact geometry can alter circuit impedance and the spatial distribution of current density, potentially influencing lesion geometry and the likelihood of achieving transmurality [11]. Prior experimental work has demonstrated that enlarging the return electrode surface area can lower circuit impedance, increase delivered current, and substantially increase lesion dimensions and transmurality in bipolar ablation. Accordingly, our predictive relationships should be interpreted in the context of this specific catheter pairing and may not directly extrapolate to configurations employing markedly different return‐electrode sizes or contact orientations.

Bhaskaran et al. [12] suggested that impedance drop is an effective indicator of lesion formation during bipolar RF application. However, they observed that increasing RF energy did not necessarily deepen lesions but rather increased the incidence of steam‐pop, particularly at settings of 20‐50 W for 60 s. Our steam‐pop model, driven by RF energy, baseline impedance, and the early impedance trajectory, parallels those observations and quantifies their joint contribution to safety.

Recent work has also underscored the value of impedance‐guided or temperature‐limited feedback to mitigate steam‐pops during high‐energy bipolar delivery. Saitoh et al. [13] achieved 100% transmurality in porcine ventricles while abolishing steam‐pops by dynamically reducing power once impedance declined beyond a preset threshold. Our data support incorporating such adaptive control: simulations excluding the 5‐s impedance drop degraded predictive accuracy by 0.17 AUC, indicating that early impedance kinetics encapsulate critical thermodynamic information not captured by static tissue parameters.

Based on our results, achieving effective bipolar RF application requires assessing the wall thickness of the target site, measuring tissue impedance, and titrating output and ablation duration accordingly. While preablation assessments such as cardiac CT or MRI are often performed to identify arrhythmogenic substrates before VT ablation, these results should guide ablation settings. Intraoperative intracardiac echocardiography should also be considered.

On the other hand, our data suggest that in order to perform safe ablation avoiding steam‐pop, it is crucial to monitor the impedance drop in the first 5 s during ablation. If this drop is excessive, discontinuation of ablation should be considered. Bhaskaran et al. [9] reported that an impedance decrement of over 26% was associated with steam‐pop. This corresponded to our results that the deprecation of impedance drops from the steam‐pop prediction model resulted in the reduction of AUC by 0.17.

Additionally, Saitoh et al. [14] reported that steam‐pop is more likely to occur when the target myocardial wall thickness is thin, consistent with our findings. They noted that the impedance drop preceding steam‐pop occurs approximately 15 s beforehand. Our study showed the ability to predict steam‐pop based on the impedance drop in the first 5 s, which is not contradictory.

**FIGURE 5:**
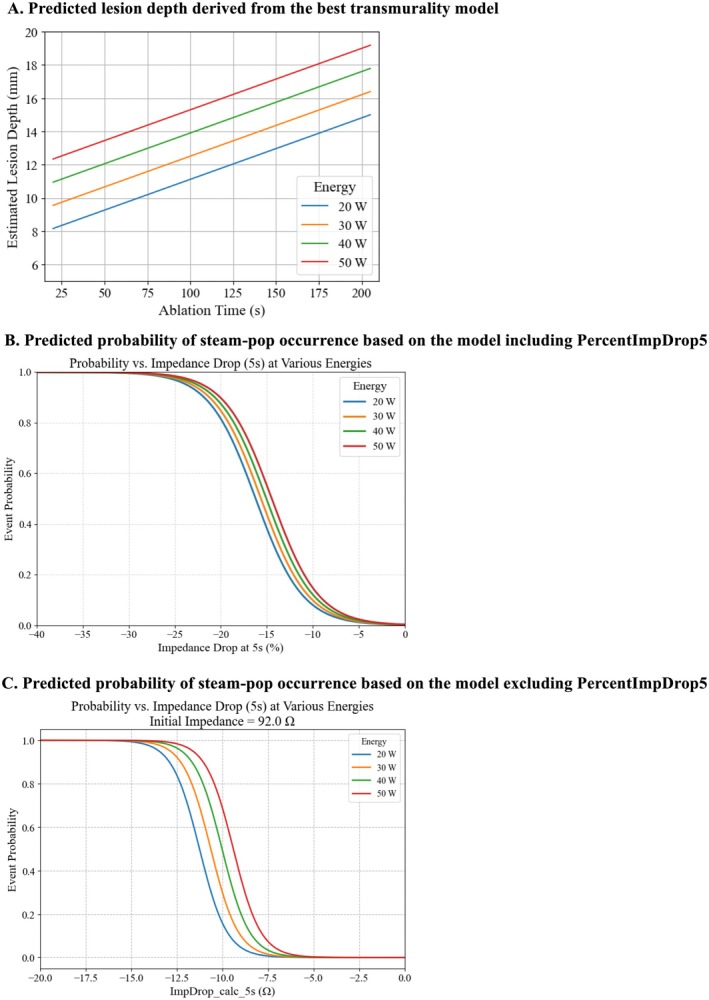
Predicted Lesion Depth and Steam‐pops. (A) Predicted lesion depth derived from the best transmurality model. The top‐performing transmurality model was rearranged to solve for lesion depth, assuming the initial impedance (Ω) relates to tissue thickness (mm) as $86.213122+0.5057449\times \text{TissueThickness}\;\left(\mathrm{mm}\right)$. Under these assumptions, higher RF energy yields deeper lesions; nevertheless, depths > 20 mm are unlikely even with a 50‐W, 200‐s application. (B) Predicted probability of steam‐pop occurrence based on the model including PercentImpDrop5. The multivariable model including PercentImpDrop5 was transformed to calculate steam‐pop probability using the sigmoid function, with initial impedance fixed at 92.0 Ω (the mean value in the training cohort). Compared to the model excluding PercentImpDrop5 (panel **C**), the difference in predicted probability across RF energy levels is smaller, reflecting the dominant contribution of PercentImpDrop5 to steam‐pop prediction. (C) Predicted probability of steam‐pop occurrence based on the model excluding PercentImpDrop5. The multivariable model excluding PercentImpDrop5 was transformed to calculate steam‐pop probability using the sigmoid function, with initial impedance fixed at 92.0 Ω (the mean value in the training cohort). The figure illustrates that at higher RF energy levels, a smaller impedance drop within the first 5 s is sufficient to reach a high probability of steam‐pop occurrence.

Applying our internally validated predictive framework, operators can predict lesion depth for any given power–duration pair (Figure 5A) and quantify the probability of steam‐pop formation over a clinically relevant power range (Figure 5B). Importantly, the time‐power look‐up curves should be interpreted within the scope of our experimental configuration (catheter pairing, contact geometry, and tested time range). In our study, extending ablation duration to 180 s achieved transmural lesions in 15‐mm tissues at ≥ 30 W, whereas 60‐s applications failed to achieve transmurality even at 50 W in 20‐mm tissues. This observation supports the concept that dose escalation through prolonged duration, rather than power increase alone, represents a viable strategy for thicker substrates. In our ex vivo setup, lesion‐depth estimates suggest that achieving an intramural depth approaching 20 mm may require prolonged delivery, and the ~200‐s estimate at 50 W represents an extrapolation beyond our longest tested application (180 s). However, transmural lesions in our dataset were achieved only up to 15 mm thickness, and predictions for 20‐mm tissue should be interpreted as conservative benchmarks rather than definitive thresholds, particularly given the nonlinear relationship between tissue thickness and transmurality. This should not be construed as a universal limitation of bipolar ablation. Prior ex vivo work using two 3.5‐mm irrigated catheters positioned perpendicular to ventricular tissue demonstrated that bipolar irrigated‐ablation can achieve transmural lesions in specimens up to 25 mm thick, although the probability of transmurality declined as thickness increased (e.g., 20–25 mm) and longer durations were suggested for very thick tissue [10]. Therefore, the present results likely provide a conservative benchmark for the specific catheter geometry and contact conditions studied here, while supporting the broader concept that thicker myocardium requires individualized titration of “dose” rather than simple power escalation.

Recent temperature‐controlled ex vivo data further support the feasibility of transmural lesion formation beyond 20 mm when the delivered dose is scaled to interelectrode distance [15]. In that study, transmurality was achieved across interelectrode distances up to 27 mm by increasing ablation index in proportion to distance, and steam pops were mitigated by temperature cutoffs (45°C at the active catheter and 55°C at the return catheter). Notably, the impedance drop within the first 5 s differed between steam‐pop and non–steam‐pop applications, which is concordant with our finding that early impedance kinetics (ImpDrop5) contribute materially to steam‐pop prediction. Together, these findings suggest practical strategies for thicker myocardial targets: prolonging application duration (or dose) rather than escalating power alone, optimizing stable contact and electrode geometry on both sides, and incorporating real‐time feedback (early impedance trajectory and/or electrode temperature) to maintain safety during longer delivery.

Moreover, when the impedance drop in the first 5 s exceeds 12.5 Ω, the estimated likelihood of a steam pop surpasses 80%, even at a power setting of 20 W.

Because the model was derived and temporally validated on an independent cohort from the same institution, its estimates already reflect variations in routine bipolar ablation practice within a real‐world setting. These data‐driven look‐up curves may therefore facilitate intraprocedural adjustment of RF energy and application time, potentially reducing both under‐treated tissue and steam‐pop‐related complications.

### 4.1 Clinical Implications

Our findings have several clinical implications, although the experiments were performed on nonbeating hearts. First, tissue thickness exceeding 20 mm may not be adequately penetrated even with 50 W bipolar ablation for 60 s; prolonged duration may be required for such thick substrates. Second, a percentage impedance drop exceeding approximately 10% within the first 5 s may signal high steam‐pop risk, prompting consideration of early termination. Third, tissues less than 10 mm thick appear to carry elevated steam‐pop risk and may warrant careful patient selection or reduced power settings when targeted with bipolar ablation.

### 4.2 Limitation

Several limitations exist in this study. Firstly, experiments were performed in ex vivo porcine myocardium rather than in beating hearts; validation in vivo is needed to build more accurate predictive models. Second, the two catheters had different tip electrode sizes (3.5‐mm THERMOCOOL SMARTTOUCH SF vs. 4‐mm tip for the FlexAbility), which may affect current density and lesion characteristics during bipolar ablation [11]. Third, contact force was controlled at 10 g only on the THERMOCOOL SMARTTOUCH SF (active) side; contact on the FlexAbility (return) side could not be quantified because it lacks a force sensor, and some variability cannot be excluded despite mechanical stabilization. Fourth, we could not evaluate catheter‐tip temperature as a predictor of steam‐pops because our single‐generator configuration connected the FlexAbility to the indifferent (return) port, where tip temperature is not recorded, and the open‐irrigated THERMOCOOL SMARTTOUCH SF thermocouple underestimates true tissue temperature [15]. Finally, our models were developed using a GLM under a specific generator/catheter/irrigation setup and may not capture nonlinear interactions. Further research is warranted to enhance the efficacy and safety of ablation procedures.

## 5 Conclusions

Our data show that accurate assessment of tissue thickness is the dominant predictor of transmural lesion formation during bipolar radiofrequency ablation. For safety monitoring, the percentage impedance drop within the first 5 s of energy delivery alone achieved an AUC of 0.89 for steam‐pop prediction, highlighting its potential as a real‐time termination criterion. Prospective studies integrating real‐time monitoring of RF parameters in the beating heart are warranted to translate these findings into clinical practice.

## Funding

The authors have nothing to report.

## Conflicts of Interest

The authors declare no conflicts of interest.

## Supporting information


**Figure S1:** Lesion formation after prolonged bipolar radiofrequency ablation. Prolonged bipolar radiofrequency applications of ≥ 30 W delivered for 180 s consistently produced transmural lesions.

**Figure S2:** Risk Borderline for Steam Pop in Development Set. Parameter combinations to the left of the oblique boundary (red‐shaded region) exceed the ≈93% steam‐pop probability predicted by our logistic model, whereas those to the right (white region) fall below this risk level. To rigorously test models' accuracy, all validation experiments were conducted under conditions immediately adjacent to the delineating boundary.

**Figure S3:** Performance of generalized linear models for predicting transmurality. A forest plot showing the area under the receiver‐operating characteristic curve (AUC) with 95% confidence intervals for each generalized linear model. The model including RF energy, RF duration, initial impedance, and tissue thickness achieved the highest AUC (0.950; 95% CI 0.908–0.992). Models containing tissue thickness consistently outperformed those without it.

**Figure S4:** Performance of generalized linear models for predicting steam‐pop occurrence. A forest plot showing the area under the receiver‐operating characteristic curve (AUC) with 95% confidence intervals for each generalized linear model. The model including RF energy and percent impedance drop in the first 5 s achieved the highest AUC (0.895; 95% CI 0.823–0.968). Models containing percent impedance drop in the first 5 s consistently outperformed those without it.

## Data Availability

The data that support the findings of this study are available on request from the corresponding author. The data are not publicly available due to privacy or ethical restrictions.
