# Supplementary figures and images for: Predicting Transmural Lesion Formation and Steam‐Pop Occurrence During Bipolar Ablation—Ex Vivo Porcine Model
Source: J Arrhythm. 2026 Apr 7;42(2):e70337. doi: 10.1002/joa3.70337 (PMC13058229; doi:10.1002/joa3.70337)

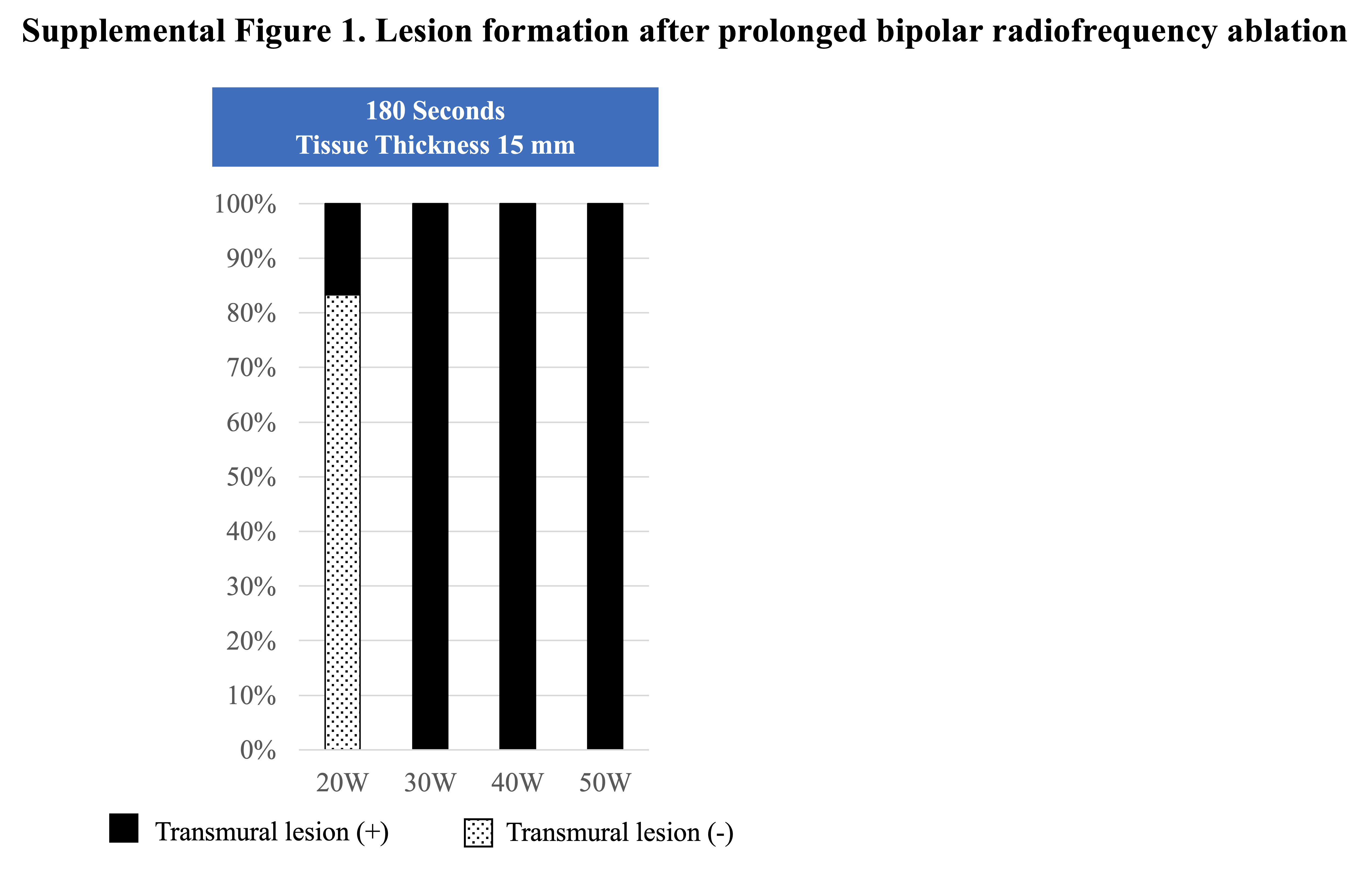

Supplement: Supplementary file 1 — Figure S1: Lesion formation after prolonged bipolar radiofrequency ablation. Prolonged bipolar radiofrequency applications of ≥ 30 W delivered for 180 s consistently produced transmural lesions. [file JOA3-42-e70337-s002.tiff]

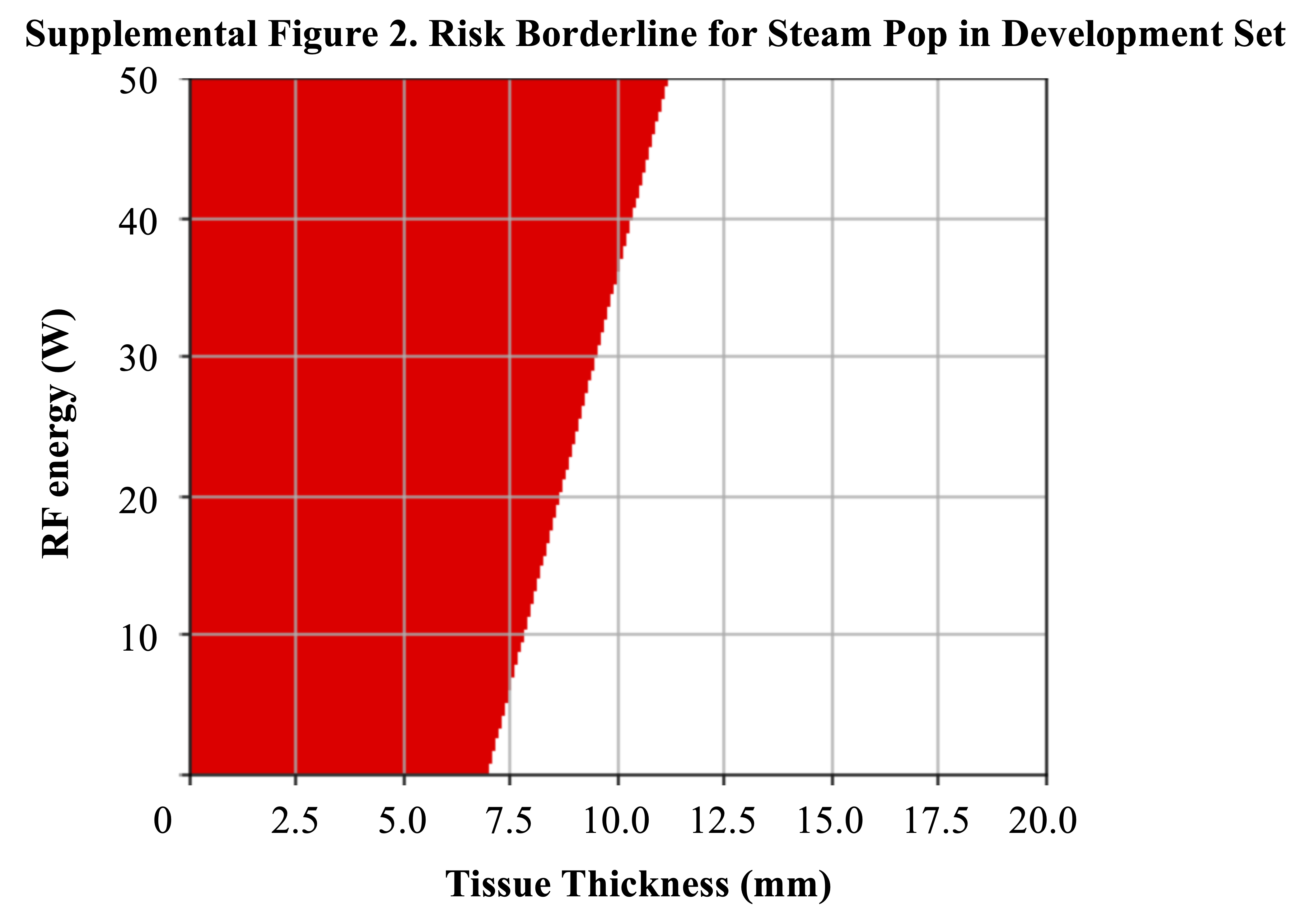

Supplement: Supplementary file 2 — Figure S2: Risk Borderline for Steam Pop in Development Set. Parameter combinations to the left of the oblique boundary (red‐shaded region) exceed the ≈93% steam‐pop probability predicted by our logistic model, whereas those to the right (white region) fall below this risk level. To rigorously test models' accuracy, all validation experiments were conducted under conditions immediately adjacent to the delineating boundary. [file JOA3-42-e70337-s004.tiff]

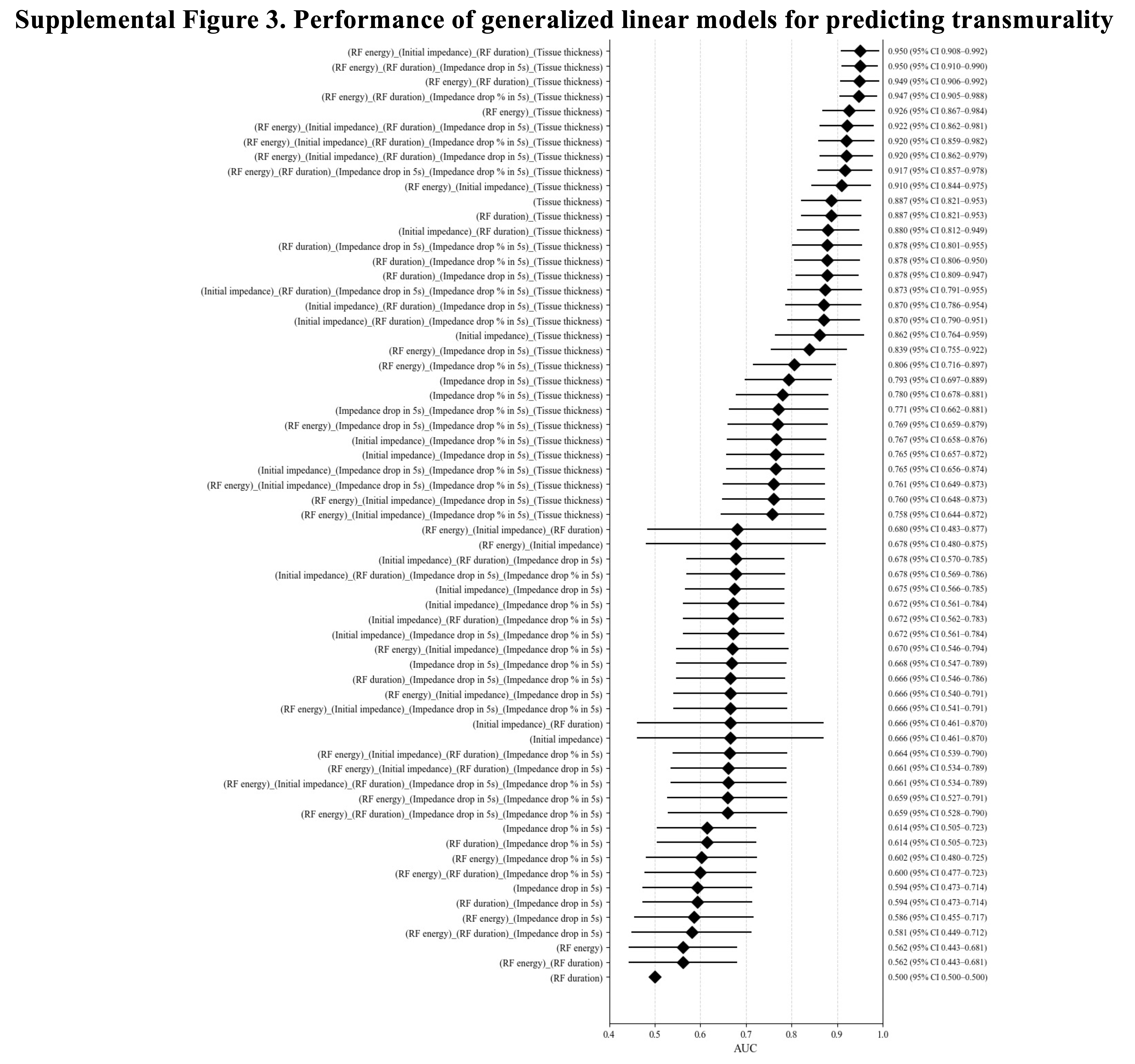

Supplement: Supplementary file 3 — Figure S3: Performance of generalized linear models for predicting transmurality. A forest plot showing the area under the receiver‐operating characteristic curve (AUC) with 95% confidence intervals for each generalized linear model. The model including RF energy, RF duration, initial impedance, and tissue thickness achieved the highest AUC (0.950; 95% CI 0.908–0.992). Models containing tissue thickness consistently outperformed those without it. [file JOA3-42-e70337-s003.tiff]

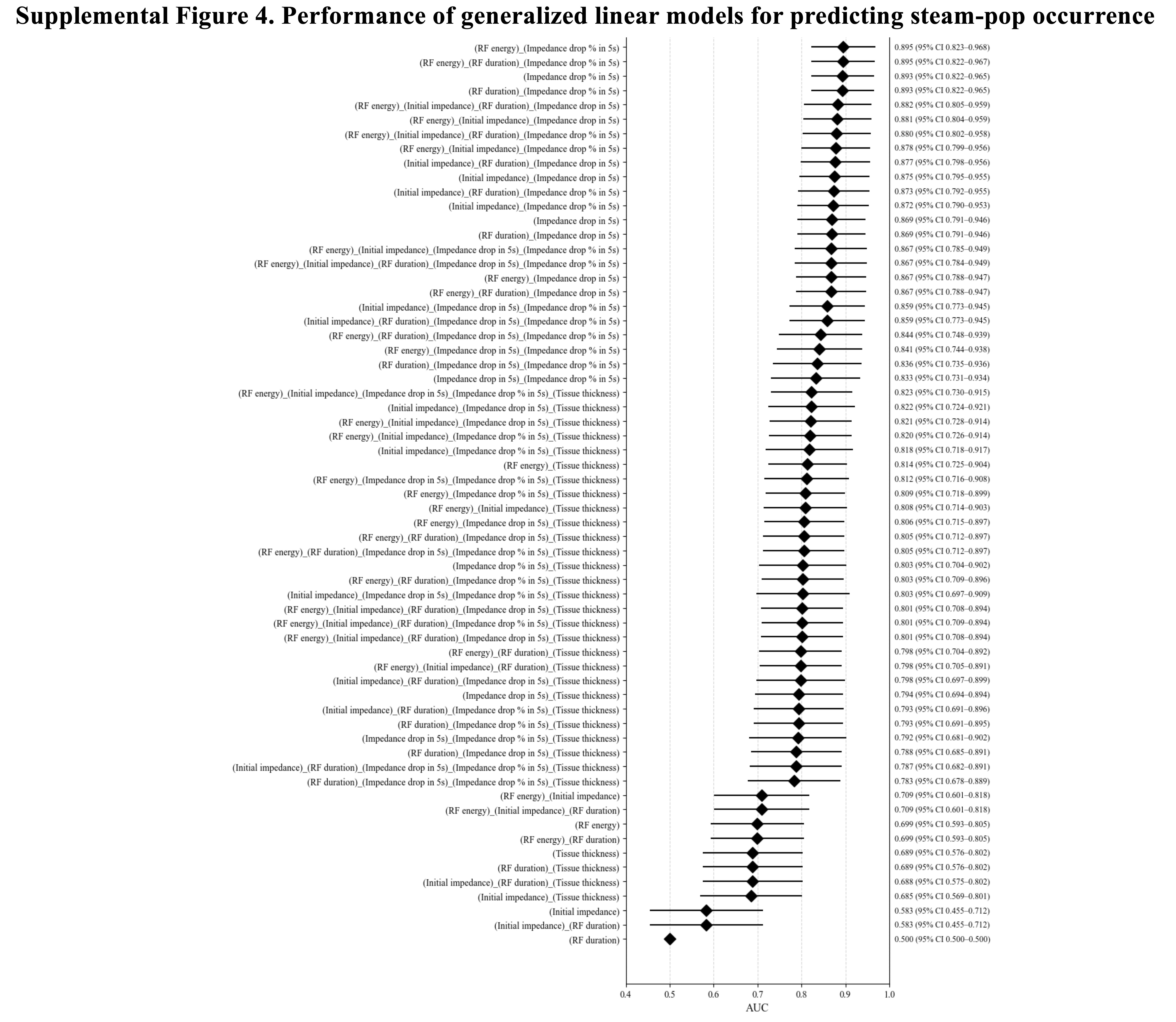

Supplement: Supplementary file 4 — Figure S4: Performance of generalized linear models for predicting steam‐pop occurrence. A forest plot showing the area under the receiver‐operating characteristic curve (AUC) with 95% confidence intervals for each generalized linear model. The model including RF energy and percent impedance drop in the first 5 s achieved the highest AUC (0.895; 95% CI 0.823–0.968). Models containing percent impedance drop in the first 5 s consistently outperformed those without it. [file JOA3-42-e70337-s001.tiff]
